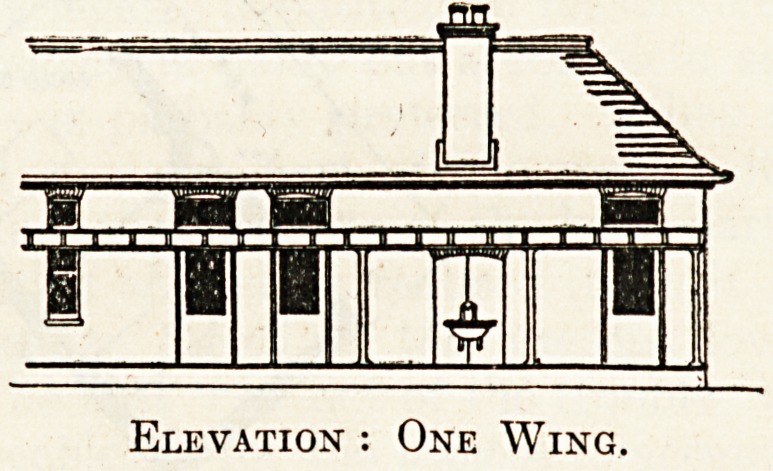# Observation Wards in Isolation Hospitals: The Cruciform Plan

**Published:** 1915-06-26

**Authors:** Christopher Chart


### HOSPITAL ARCHITECTURE AND CONSTRUCTION.

### Observation Wards in Isolation Hospitals.

### THE CRUCIFORM PLAN.

By CHRISTOPHER CHART, F.S.I., Dip.San.Soc., M.S.A.

For some years attempts have been made to find a solution of the difficulties experienced in hospitals in providing isolation accommodation for single cases of infectious disease, for cases of mixed infection or of doubtful diagnosis. These cases render the other beds in the ward for the time unavailable, and necessitate the services of the nurses being confined to attendance on one patient.

#### Three Types of Design.

The adoption of the "Barrier system" of nursing has not proved completely successful. By this method the beds are not actually separated by any physical barrier other than a cord placed round the bed, beyond which the patient is not allowed, and which indicates the necessity for special precautions by the nursing staff. As an aid to this system three types of ward arrangement have been adopted varying in the degree of aerial separation between the patients—viz.:

1. The division of a large ward into cubicles, by partitions not reaching to the ceiling, as at the South-Western Hospital of the Metropolitan Asylums Board. This system does not appear to have been wholly successful in cases of small-pox, chicken-pox, septic scarlet fever, and in the acute stages of measles.

2. An arrangement of compartments each separated from any other by partitions reaching to the ceiling, the compartments entered from a common corridor. This plan was adopted at East Ham and in the Pasteur and Sick Children's Hospitals at Paris.

3. Completely separated compartments, each separately entered from the open air under a verandah, which is the type of plan dealt with in this article. It may be noted, in passing, that the partitions between the compartments are wholly or partially of glass to facilitate supervision.

#### The Walthamstow Hospital Plan.

The first hospital block of type 3 erected by a local authority was that at the Walthamstow Hospital opened in 1906. Externally, it appears to be a pavilion of the usual type—*i.e.*, a duty-room with one large ward on either side, but with a verandah running completely round the building. Detached w.c.s, sink, and bathrooms entered from the end verandahs. Internally, each large ward is divided by longitudinal and transverse glazed partitions into six compartments, aerially distinct from each other, and entered from the verandah by separate doors. Each compartment has a window of the usual hospital type.

Borough of Cambridge Observation Wards.

#### The Ventilation Question.

Owing to their back-to-back arrangement special means of securing through ventilation have to be arranged. For this purpose an air duct is brought from an opening near the ground level beyond the verandah on the opposite side of the block to a grating in the floor, the duct passing under the floor of the adjoining compartment. Each compartment has also a ceiling ventilator.

### EVOLUTION OF THE CRUCIFORM PLAN.

In 1908-9 the Croydon Rural District Council were proposing to further extend their isolation hospital at Beddington Corner, which had been designed by the writer's firm (1896, and extensions 1904), including, amongst other additions, an observation ward on the lines of that recently erected at Walthamstow, and preliminary plans were prepared for that purpose. Before formal application for permission to borrow money was made, the plans were submitted to the Local Government Board's officers. They suggested that as an alternative to the air ducts under the floors, as at Walthamstow, cross-ventilation might be obtained by an arrangement of oblique shafts in the roof space, crossing the adjoining compartment, to open on the opposite side of the ridge of the roof, and a plan was worked out embodying this suggestion.

SECTION.

The construction of these oblique shafts presented certain structural difficulties; they rendered the roof-space less accessible for reaching gas and water, services which could otherwise have been conveniently placed there, while the lower side of the oblique shaft would have given a lodgment for dust, and from its position above the ceiling level of the ward could not have been readily cleaned. In addition to these objections, it appeared to the writer that the same principles which have led to the prohibition of back-to-back houses should be applied to hospital buildings, and that any further improvement of this type of building should be based on a plan from which the back-to-back arrangement was eliminated.

### THE CRUCIFORM PLAN AT CROYDON.

An alternative plan was accordingly prepared embodying these suggestions in a cruciform building, and the two alternatives were discussed with the late Dr. Franklin Parsons, of the Local Government Board, with the result that the cruciform plan was adopted, and the building erected to that design.

This pavilion, which was opened in the autumn of 1911, has a large central octagonal duty-room and four wings pointing N.W., S.W., S.E., and N.E., each of which contains three single-bed compartments entirely separated from one another by plate-glass partitions. The compartments are entered separately from open verandahs. The verandahs of the N.W. and S.W. wings, which are used for females, are continuous with each other, meeting on the W. side at an angle in which is placed the annexe containing the w.c. and slop-sink. The N.E. and S.E. wings, used for males, are similarly arranged, with a verandah on the E. side. Windows on either side of each wing provide cross-ventilation. Special precautions are taken in nursing to avoid risk of cross-infection. A separate overall for use of the nurse hangs in the verandah outside each compartment, and a pedal-action lavatory is placed in the verandah of each wing, so that she may wash her hands between attending one patient and another. A portable bath stands in each verandah, which is wheeled into the wards when required. The pavilion at Beddington Corner has been in continuous use since its erection, from eighty to ninety patients being nursed therein annually, and up to the present no case of cross-infection has occurred.

### THE NEW CAMBRIDGE HOSPITAL.

In 1913 the Cambridge Borough Council were proposing to extend their hospital by the addition of two pavilions, one of which was to be arranged on the separate compartment system. After visiting and inspecting other buildings of this type it was decided to adopt the cruciform plan as erected at Beddington Corner. The new buildings are now complete and were, as reported in an earlier issue of THE HOSPITAL, opened on May 12 last.

One of the new pavilions is of the ordinary type and is to be used for scarlet fever. It has two large wards for six beds each and two single-bed wards, and a convalescent or day room placed over the duty room. This pavilion has wide verandahs on both sides, open in front, the ends filled with glazed screens so that the beds can be wheeled out for open-air treatment if required.

ELEVATION: ONE WING.

The other pavilion, based on the Beddington Corner plan, is a cruciform building and the general arrangements are shown on the plan reproduced here. The principal points of difference between this and the earlier building are that the wings are slightly more inclined to the N. and S. respectively so as to give more space for the w.c. and slop-sink annexe, to which has been added a small bathroom.

The provision of a bathroom affords accommodation for patients bathing, provided precautions are taken in passing to and fro and in cleaning after use to prevent infecting other patients. This arrangement obviates the necessity of keeping the baths on the verandahs. The baths are mounted on wheels so that they can also be used in the wards.

The pedal lavatories, which at Beddington Corner are placed on the flat surface of the wall projecting into the verandahs, are at Cambridge placed in the re-entering angle of the corner fireplaces of the wards. In this position they do not obstruct the verandahs and are more protected from frost, which has given some trouble in the earlier building.

The coal bunkers shown on the plan are filled from outside. They are of hopper shape with door at the bottom into the ward at floor level, and have been found to effect a saving in labour. These were made first for a new pavilion erected some ten years ago to avoid the necessity of the hospital porter having to carry coals into the wards. They were constructed of galvanised iron and were satisfactory in working, but too much noise was made in filling them. In order to meet this objection the body of the hopper is now built in reinforced concrete.

The plan and a full description of the Beddington Corner Hospital, now transferred to the Croydon Rural and Merton Joint Hospital Board, will be found in the late Dr. Franklin Parsons' Report on Isolation Hospitals, issued as a Supplement to the Annual Report of the Local Government Board, 1910-11. Chapter VI. deals especially with compartment and cubicle blocks and with nursing precautions to prevent cross-infection. The latter are rather outside the scope of an article dealing with the planning of the pavilions, though they must be considered and provided for by the architect. However well designed the building, successful results can only be obtained by efficient nursing under the superintendence and directions of the physician.

## Figures and Tables

**Figure f1:**
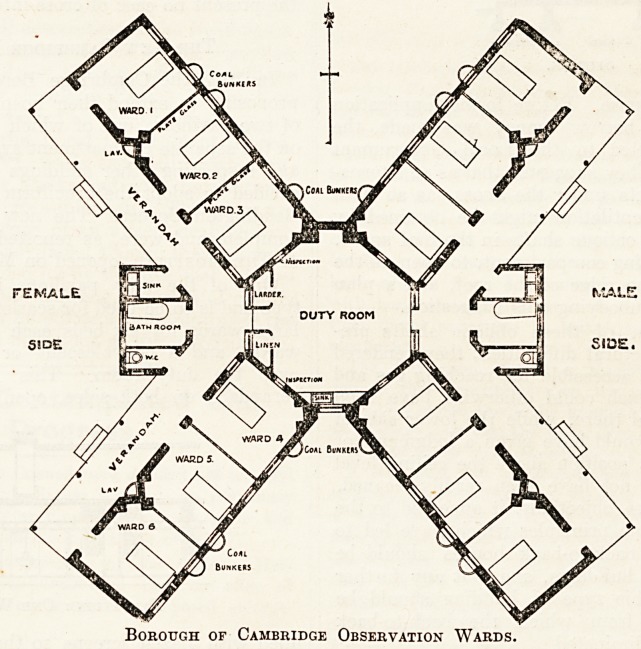


**Figure f2:**
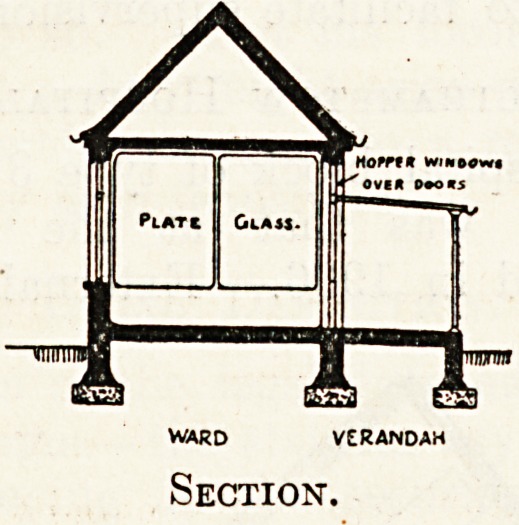


**Figure f3:**